# Grating visual acuity impairment assessed by sweep visually evoked potentials in children with optic pathway tumors unable to perform optotype acuity tests

**DOI:** 10.5935/0004-2749.20210022

**Published:** 2025-02-02

**Authors:** Patrícia de Freitas Dotto, Adriana Berezovsky, Andrea Maria Cappellano, Nasjla Saba da Silva, Paula Yuri Sacai, Daniel Martins Rocha, Erica Pinheiro de Andrade, Frederico Adolfo B. Silva, Solange Rios Salomão

**Affiliations:** 1 Laboratório de Eletrofisiologia Visual Clínica, Departamento de Oftalmologia e Ciências Visuais, Escola Paulista de Medicina, Universidade Federal de São Paulo, São Paulo, SP, Brazil; 2 Programa de Neuro-Oncologia, Instituto de Oncologia Pediátrica, Grupo de Apoio ao Adolescente e à Criança com Câncer, Universidade Federal de São Paulo, São Paulo, SP, Brazil

**Keywords:** Visual disorders, Evoked potentials, visual, Visual acuity, Visual pathways, Optic nerve glioma, Child, Transtornos da visão, Potenciais evocados visuais, Acuidade visual, Vias visuais, Glioma do nervo óptico, Criança

## Abstract

**Purpose:**

To determine visual impairment due to optic pathway tumors in children unable to perform recognition acuity tests.

**Methods:**

Grating visual acuity scores, in logMAR, were obtained by sweep visually evoked potentials (SVEP) in children with optic pathway tumors. The binocular grating visual acuity deficit was calculated by comparison with age-based norms and then assigned to categories of visual impairment as mild (from 0.10 to 0.39 logMAR), moderate (from 0.40 to 0.79 logMAR), or severe (≥0.80 logMAR). Interocular differences were calculated by subtraction and considered increased if >0.10 logMAR.

**Results:**

The participants were 25 children (13 boys; mean ± SD age, 35.1 ± 25.9 months; median age, 32.0 months) with optic pathway tumors (24 gliomas and 1 embryonal tumor), mostly located at the hypothalamic-chiasmatic transition (n=21; 84.0%) with visual abnormalities reported by parents (n=17; 68.0%). The mean grating acuity deficit was 0.60 ± 0.36 logMAR (median, 0.56 logMAR). Visual impairment was detected in all cases and was classified as mild in 10 (40.0%), moderate in 8 (32.0%), and severe in 7 (28.0%) children, along with increased interocular differences (>0.1 logMAR) (n=16; 64.0%). The remarkable ophthalmological abnormalities were nystagmus (n=17; 68.0%), optic disc cupping and/or pallor (n=13; 52.0%), strabismus (n=12; 48.0%), and poor visual behavior (n=9; 36.0%).

**Conclusion:**

In children with optic pathway tumors who were unable to perform recognition acuity tests, it was possible to quantify visual impairment by sweep-visually evoked potentials and to evaluate interocular differences in acuity. The severity of age-based grating visual acuity deficit and interocular differences was in accordance with ophthalmological abnormalities and neuroimaging results. Grating visual acuity deficit is useful for characterizing visual status in children with optic pathway tumors and for supporting neuro-oncologic management.

## Abstract (pt)

**Objetivo:**

Determinar o grau de deficiência visual em crianças com tumores da via óptica incapazes de informar a acuidade visual de reconhecimento.

**Método:**

A acuidade visual de grades, em logMAR, foi estimada por potenciais visuais evocados de varredura em crianças com tumores das vias ópticas. O déficit da acuidade visual de grades binocular foi calculado em relação ao valor mediano normativo esperado para a idade e a deficiência visual, classificada como leve (0,10 a 0,39 logMAR), moderada (0,40 a 0,79 logMAR) ou grave (≥0,80 logMAR). Diferenças inter-oculares foram calculadas por subtração e consideradas aumentadas se >0,10 logMAR.

**Resultados:**

Foram avaliadas 25 crianças (13 meninos; média de idade ± DP=35,1± 25,9 meses; mediana=32,0 meses) com tumores da via óptica (24 gliomas e 1 tumor embrionário) localizados particularmente na transição hipotalâmico-quiasmática (n=21; 84,0%) e com anormalidades visuais detectadas pelos pais (n=17; 68,0%). A média do déficit da acuidade de grades foi 0,60 ± 0,36 logMAR (mediana=0,56 logMAR). Observou-se deficiência visual leve em 10 (40,0%), moderada em 8 (32,0%) e grave em 7 (28,0%), além de aumento da diferença interocular da acuidade visual (n=16; 64,0%). As principais alterações oftalmológicas encontradas foram: nistagmo (n=17; 68,0%), aumento da escavação do disco óptico e/ou palidez (n=13; 52,0%), estrabismo (n=12; 48,0%) e comportamento visual pobre (n=9; 36,0%).

**Conclusão:**

Em crianças com tumor da via óptica e incapazes de responder aos testes de acuidade visual de reconhecimento, foi possível quantificar deficiência visual por meio dos potenciais visuais evocados de varredura e avaliar a diferença interocular da acuidade visual de grades. A gravidade do déficit da acuidade visual de grades relacionado à idade e a diferença interocular da acuidade visual de grades foram congruentes com alterações oftalmológicas e neuroimagem. O déficit da acuidade visual de grades foi útil à caracterização do estado visual em crianças com tumores da via óptica e ao embasamento da assistência neuro-oncológica.

## INTRODUCTION

Brain tumors are common space-occupying pediatric neoplasms, with an incidence varying from 1.12 to 5.14/100,000 according to age range, histologic subtype, and country^(1,2)^. According to the Central Brain Tumor Registry of the United States statistical report, gliomas followed by embryonal tumors are prevalent in patients under 14 years old, whereas older patients usually present with pineal tumors followed by gliomas^(3)^. Activation and overexpression of proto-oncogenes, as well as loss or inactivation of tumor suppressor genes, are the main biological mechanisms underlying the neoplastic changes^(4)^. In the course of disease development, several neuronal routes can be disrupted. Vision may be progressively disturbed by optic pathway compression, tumor invasion, or even surgical intervention^(5,6)^, leading to irreversible blindness, considered as one of the most dramatic neurological sequelae in survivors^(7)^.

Because the visual system in children is still developing, even temporary interruption of normal visual input can lead to a permanent decrease in vision from amblyopia^(8)^. Mild visual disturbances may be hardly recognizable or misinterpreted as benign ophthalmic morbidities, delaying the diagnosis in small children^(9)^.

Visual acuity (VA) testing using optotypes such as Snellen charts, Early Treatment Diabetic Retinopathy Study (ETDRS) charts, or Lea symbols is usually the gold standard for assessment of visual function in children with brain tumors^(10)^. These tests evaluate recognition acuity, which is the ability to recognize and name symbols (optotypes) presented in table form or designed, built on the principles of Snellen. The optotypes must be larger than the detection limit of the subject. A limitation of the method is that it is difficult to apply in preverbal, uncooperative, or cognitively impaired children who are unable to perform recognition tasks^(11)^. Therefore, alternative methods for visual function assessment may be required^(12-14)^.

An alternative method of evaluating VA is the adoption of resolution acuity techniques. Resolution acuity is measured by the smallest angle of separation between critical elements of a standardized stimulus composed of pairs of points, grids, or chess patterns that an individual can discriminate. Resolution acuity, such as grating acuity, can be measured subjectively by Teller acuity cards or objectively by the sweep visually evoked potentials (SVEP) technique^(12,13)^. Both techniques have been clinically used in recent decades as ancillary tests to evaluate subjects who are unable to perform recognition VA. The advantages of SVEP are that it provides an objec tive, reliable, and rapid (requiring only a 10-second trial) estimation of VA. It has become a precise method of evaluating uncooperative children^(14)^. Moreover, SVEP is based on cortical threshold responses, which reduces the influence of the examiner on VA results.

Although SVEP has been employed to monitor VA during normal development^(13)^ and in severe clinical conditions associated with blindness^(15,16)^, few studies have employed SVEP to investigate visual function in patients with brain tumors^(17,18)^. Obtaining reliable data on visual function from a sick child with a brain tumor is difficult, but maximizing this information can influence future treatment decisions^(19)^.

The purpose of this study was to determine grating visual acuity deficits (GVAD) and visual impairment in children with optic pathway tumors who were unable to undergo recognition acuity testing due to young age, developmental delay, or neurological sequelae. We believe our results can provide useful information to hasten suspected diagnoses and neuro-oncologic management, aiming to achieve optimal visual outcomes in survivors.

## METHODS

Children diagnosed with optic pathway tumors were referred to the Clinical Electrophysiology of Vision Laboratory of the Universidade Federal de São Paulo (UNIFESP) for grating acuity measurement by SVEP between May 2002 and May 2018. The study followed the tenets of the Declaration of Helsinki and its later amendments. Institutional Review Board approval was obtained from the Committee on Ethics in Research of UNIFESP.

The inclusion criteria were children 8 years old or younger, unable to perform recognition VA testing, with an unequivocal diagnosis of optic pathway tumors determined by pediatric neuro-oncology experts and classified according to the 2016 World Health Organization Classification of Tumors of the Central Nervous System^(19)^. The exclusion criteria were infectious diseases, congenital or drug-induced cataract, structural abnormalities affecting the visual axis, and abnormal macular aspect.

Ophthalmic assessment of children with optic pathway tumors included visual fixation, visual pursuit, eye alignment (Hirschberg, Krimsky, or cover test), presence of nystagmus, external examination of the eyes, and fundus examination. All children were awake, alert, and wearing their glasses when required. Symptoms, tumor onset, tumor location on neuroimaging, and tumor management were also noted.

Binocular and monocular grating visual acuity (GVA) measurements were performed using the PowerDiva (digital infant vision assessment) SVEP system (Smith- Kettlewell Eye Research Institute, San Francisco, CA, USA)^(14)^. The SVEP system is composed of two interfacing Macintosh G3 computers: the ‘‘host’’ computer, in charge of stimulus trial parameters and analysis of visually evoked potentials (VEP), and the ‘‘video’’ computer, linked to the monitor where stimuli are shown to the subject. The SVEP procedure was performed in a dark room with the child seated on their parent’s lap or in a wheelchair. SVEP was recorded only when the subject was alert and fixating the stimuli. To ensure attention, small toys were dangled over the center of the display. The total testing time, including setup and rest breaks, typically lasted from 10 to 30 minutes, depending on the subject’s age and cooperation.

The electroencephalogram (EEG) was recorded from two bipolar active placements (O_1_ and O_2_) with a ground electrode positioned 1 cm above the inion on the midline (O_z_), in accordance with the 10-20 System. A reference electrode was placed on the vertex (C_z_). Electrodes (Grass Gold Disc Electrodes-E6GH, Astro-Med Inc. USA) were attached to the scalp with electrode cream after cleansing the scalp with abrasive paste and cotton pads. A headband (3M Coban self-adherent Wrap 1581) was used to keep the electrodes in place. The stimuli were phase-reversal sine-wave gratings presented on a high-resolution 17-inch monochromatic video monitor (M20DCD4RE-Richardson Electronics^®^ Ltd. USA) at a fixed contrast (80%) and mean luminance of 142.35 cd/m^2^. The test stimulation field varied from 52° × 65° (for 30-cm distance) to 11° × 14° (for 150-cm distance), for both vertical and horizontal monitor lengths. For all tests, the spatial frequency was swept (from 0.1 to 30 cycles per degree) by the viewing distance (30 to 150 cm) with temporal modulation of 6.6 Hz. Ten linearly spaced spatial frequencies were presented at a rate of 1/s, starting at a low spatial frequency. The gratings were vertically oriented, except in cases of horizontal nystagmus^(13-16)^.

The recordings were adaptively filtered (bandpass) in real time (sampling rate, 397 Hz) to isolate the VEP. The potential differences were amplified (Neurodata Acquisition System P15, Grass Instrument Co., USA) (gain, 10,000; - 3 db cutoff at 1 and 100 Hz). Three to 15 repetitions of the sweep were obtained and vector averaged. The amplitude and phase of the first (6 Hz) and second (12 Hz) harmonics of the stimulus frequency were calculated for each channel by discrete Fourier transform(13-16).

**Figure 1:**
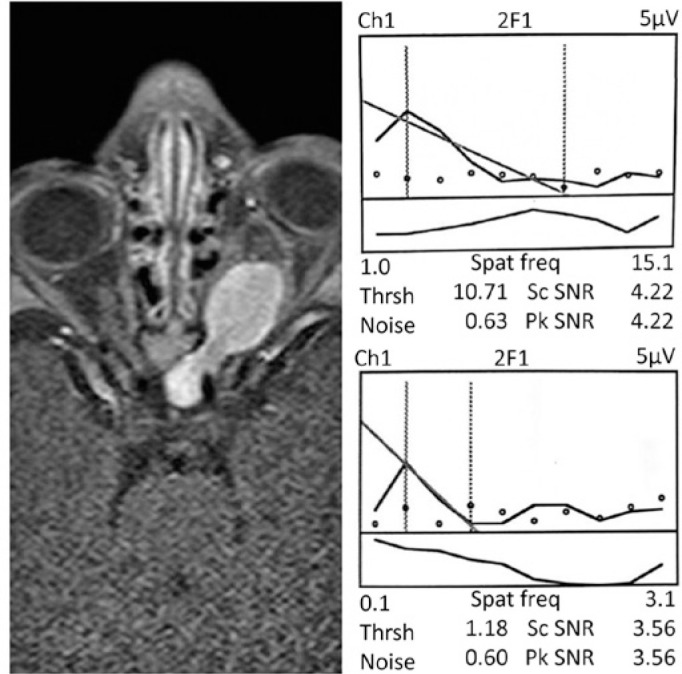
Representative sweep visually evoked potential (SVEP) response from the better-seeing eye (BSE) and the worse-seeing eye (WSE) (right panel) and orbital magnetic resonance imaging (MRI) (left panel) from subject S01. Right panel: SVEP from channel 1 (O1-Cz). Dots represent noise registered for each of 10 linearly spaced spatial frequencies presented (from 1.00 to 15.10 cycles/degree presented at 80 cm for the right eye and from 0.1 to 3.01 cycles/degree presented at 30 cm for the left eye); grating acuity was estimated by linear fit and extrapolation to zero amplitude, i.e., the value at which the regression line touches the axis of spatial frequencies; the final acuity scores were thresholds of 10.71 cycles/degree, equal to 0.44 logMAR (or 20/55 Snellen fraction) for the BSE and 1.18 cycles/ degree, equal to 1.40 logMAR (or 20/510). The signal-tonoise ratio was 4.22 for the right eye and 3.56 for the left eye. Left panel: Enhanced axial T1-weighted MRI (960/20 [repetition time msec/echo time msec]) shows high signal intensity around the left optic nerve from its intraorbital portion to the chiasm, whereas the intraorbital portion of the right optic nerve is normal. The reduced grating visual acuity (GVA) in the right eye (BSE) is probably due to effects on the optic nerve at the chiasm level. Abbreviations: Ch= channel; 2F1s= harmonic of the stimulus frequency; Spat Freq= spatial frequency; Thrsh= grating acuity threshold (cycles/ degree); SNR= signal-to-noise ratio; Sc SNR= maximum SNR within the cursors (dotted lines) that define the data used to estimate threshold; Pk SNR= maximum SNR at peak mean amplitude in the record.

Grating acuity was estimated with an automated algorithm that performed a linear fit and extrapolation to zero amplitude for the final descending limb of the function relating each VEP amplitude (from the second harmonic) to a linear spatial frequency (Figure 1). A signal-to-noise ratio (SNR) at a peak of 3:1 was required and calculated as the ratio of the power at stimulus frequency to the mean power at frequencies ± 2 Hz, corresponding to a false alarm rate of 0.4 %, ensuring an adequate protection level when combined with the phase consistency criteria. In all cases, two thresholds (one for O_1_ and another for O_2_) were obtained. The final acuity score was calculated in logMAR (logarithm of the minimum angle of resolution) using the results in cycles per degree of visual angle of the better threshold channel with the highest SNR^(13-16)^.

The eye with the better grating acuity measurement was classified as the better-seeing eye (BSE) and the fellow eye as the worse-seeing eye (WSE). If similar acuities were found in both eyes, the BSE was randomly assigned using the RANDARRAY function of MSExcel Software considering 0 as the right eye and 1 as the left eye. Visual acuities of light perception (previously confirmed by transient flash visually evoked potentials recordings) were assigned as 3.0 logMAR^(5)^. Monocular measures were employed to calculate interocular acuity differences (IAD) by subtraction; the IAD was classified as increased if >0.1 logMAR.

Binocular GVAD was obtained by subtracting binocular GVA scores from the age-related median norm^(14)^. Visual impairment was categorized as mild (0.39 ≥ GVAD ≥0.10 logMAR), moderate (0.79 ≥ GVAD ≥0.40 logMAR), or severe (GVAD ≥0.80 logMAR)^(15)^. To validate the method, the cutoff value of 0.10 logMAR in GVAD for normal acuity was previously established as a function of GVA variation from a group of 10 healthy children aged 8 years or younger (6 girls; mean ± SD age, 75.0 ± 15.6 months; median age, 76.0 months), presenting with recognition VA equal to 0.00 logMAR (20/20 Snellen fraction) and normal ophthalmological examination (normal fundus, normal ocular motility, normal Titmus stereo test equal to 40”, preserved pupillary reflexes, best-corrected VA 4-m ETDRS chart equal to or better than zero logMAR, and spherical equivalent of the refractive status from - 6.00 to + 6.00 diopters). In this control group, the monocular mean GVAs were 0.04 ± 0.02 logMAR (median, 0.04 logMAR) and 0.05 ± 0.03 logMAR (median, 0.06 logMAR) for BSEs and WSEs, respectively (Table 1).

**Table 1:** Demographics, recognition (Snellen optotypes), and grating visual acuity (GVA) measured by the sweep visually evoked potentials (SVEP) technique, both expressed in the logarithm of the minimum angle of resolution (logMAR), and the spherical equivalent of the refractive error, in diopters (D), from the better-seeing eyes (BSE) and the worse-seeing eyes (WSE) of 10 healthy children

Individual	Sex/age (mo)	BSE		WSE	
		Snellen optotypes (logMAR)	GVA (logMAR)	Snellen optotypes (logMAR)	GVA (logMAR)
C01	F/52	0.00	0.03	0.00	0.04
C02	F/55	0.00	0.02	0.00	0.06
C03	M/60	0.00	0.04	0.00	0.05
C04	M/70	0.00	0.01	0.00	0.01
C05	F/72	0.00	0.05	0.00	0.06
C06	F/80	0.00	0.01	0.00	0.03
C07	F/85	0.00	0.01	0.00	0.01
C08	M/88	0.00	0.06	0.00	0.08
C09	F/93	0.00	0.06	0.00	0.07
C10	M/95	0.00	0.07	0.00	0.09

### Statistical analysis

An unpaired *t*-test was performed to compare GVAD between boys and girls with optic pathway tumors after the normality test (Shapiro-Wilks). The correlation between the age of tumor onset and GVAD was investigated by the Pearson correlation test. Statistical significance was established at p≤0.05.

## RESULTS

The participants were 25 children (13 boys) with ages ranging from 3 to 95 months (mean ± SD, 35.1 ± 25.9 months; median, 32.0 months), with optic pathway tumors and unable to perform recognition acuity tests. The age of tumor onset ranged from birth to 36 months (mean ± SD, 10.8 ± 11.3 months; median, 6.0 months). The lesions were classified as diffuse astrocytic and oligodendroglial tumors (n=13), including 6 lowgrade gliomas, 6 astrocytomas, and 1 glioblastoma; other astrocytic tumors (n=10), including 9 pilocytic astrocytomas and 1 pilomyxoid astrocytoma; neuronal and mixed neuronal-glial tumors (n=1): 1 desmoplastic infantile ganglioglioma and embryonal tumors (n=1):1 atypical teratoid/rhabdoid tumor.

Optic pathways were affected in all patients, mainly at the hypothalamic -chiasmatic transition (n=21; 84.0%). The mechanism of optic pathway disturbance by tumors was inward growth in 11 children (44.0%), secondary compression by raised intracranial pressure or mass effect in 9 children (36.0%), and both mechanisms in 5 children (20.0%). Oncologic management varied as a function of tumor type and location, requiring both single and combined therapies, including observation, chemotherapy, and/or tumor resection, and associated procedures, such as ventriculoperitoneal shunting (VPS), autologous bone marrow transplantation, and external radiation therapy. A complete description of each case, including tumor type, tumor location, effects of the tumor on the visual system, and treatment, can be found in table 2. Ocular findings and VA measurements for each participant are described in table 3.

**Table 2:** Demographics, tumor type, tumor location, suspected mechanism of effect on optic pathway, and treatment of 25 children with optic pathway tumors according to visual impairment category

Patient	Sex		Age (mo)		Tumor onset (mo)		Tumor type	Tumor location				Tumor effect on visual system	Treatment
								**Mild visual impairment (n=10)**					
**S01**	M		12		3		Astrocytoma	Hypothalamic/chiasmatic				Inward growth	Chemotherapy
**S02**		M		13		0	Pilocytic astrocytoma			Hypothalamic/chiasmatic	Mass effect		Chemotherapy + resection
**S03**		F		15		3	Pilocytic astrocytoma			Optic tract (left)	Inward growth		Observation
**S04**		F		32		0	Glioblastoma			Occipital lobe	Inward growth		Chemotherapy + resection
**S05**		M		43		31	Astrocytoma			Hypothalamic/chiasmatic	Inward growth		Observation
**S06**		F		50		36	Low-grade glioma			Hypothalamic/chiasmatic	Inward growth + raised ICP		Chemotherapy + VPS
**S07**		F		65		12	Pilocytic astrocytoma			Hypothalamic/chiasmatic	Inward growth + raised ICP		Chemotherapy + VPS
**S08**		M		79		24	Pilocytic astrocytoma			Hypothalamic/chiasmatic	Mass effect + raised ICP		Resection + VPS
**S09**		M		94		4	Astrocytoma			Hypothalamic/chiasmatic	Mass effect + raised ICP		Chemotherapy + resection + VPS
**S10**		M		95		36	Low-grade glioma			Hypothalamic/chiasmatic	Inward growth		Observation
							**Moderate visual impairment (n=8)**						
**S11**		M		3		1	Teratoid/rhabdoid			Hypothalamic/chiasmatic	Mass effect		Resection
**S12**		F		8		5	Pilocytic astrocytoma			Hypothalamic/chiasmatic	Inward growth + raised ICP		Chemotherapy + VPS
**S13**		F		9		0	Low-grade glioma			Hypothalamic/chiasmatic	Inward growth		Observation
**S14**		F		10		6	Pilocytic astrocytoma			Hypothalamic/chiasmatic	Inward growth		Chemotherapy
**S15**		F		17		6	Astrocytoma			Hypothalamic/chiasmatic	Mass effect + raised ICP		Chemotherapy + resection
**S16**		F		36		6	Low-grade glioma			Hypothalamic/chiasmatic	Inward growth		Chemotherapy
**S17**		F		40		5	Astrocytoma			Hypothalamic/chiasmatic	Inward growth		Chemotherapy + EVP
**S18**		M		54		21	Pilocytic astrocytoma			Hypothalamic/chiasmatic	Mass effect + raised ICP		Chemotherapy + resection + VPS
							**Severe visual impairment (n=7)**						
**S19**		F		18		6	Low-grade glioma		Hypothalamic/chiasmatic		Inward growth + raised ICP		Chemotherapy + VPS
**S20**		M		20		14	Pilocytic astrocytoma		Hypothalamic/chiasmatic		Mass effect		Chemotherapy + resection
**S21**		M		26		6	Desmoplastic infantile ganglioglioma		Occipital lobe		Mass effect		Resection
**S22**		F		26		22	Pilocytic astrocytoma		Hypothalamic/chiasmatic		Inward growth + raised ICP		Chemotherapy + VPS
**S23**		M		33		6	Low-grade glioma		Hypothalamic/chiasmatic		Inward growth		Chemotherapy
**S24**		M		37		18	Astrocytoma		Hypothalamic/chiasmatic		Mass effect + raised ICP		Chemotherapy + resection + VPS
**S25**		M		42		0	Pilomyxoid astrocytoma		Lateral geniculate body (right)		Inward growth		Resection

ICP= intracranial pressure; VPS= ventriculoperitoneal shunting.

**Table 3:** Demographics, clinical features, grating visual acuity (GVA) scores, and visual deficits in 25 children with optic pathway tumors according to visual impairment category

Patient	Sex/age (mo)		Clinical presentation	Seizures		Ocular motility GVA OU and nystagmus Fundus (logMAR)			GVA deficit (logMAR)		GVA BSE (logMAR)		GVA WSE (logMAR)		IAD (logMAR)	
						**Mild visual impairment (n=10)**										
**S01**	M/12		Proptosis	No		XT LE Cupping WSE 0.44			0.11		0.44		1.40		**0.96**	
**S02**		M /13	NF1 and shaking eyes		No	ORTHO/Nys	NA	0.63		0.30		0.61		0.63		0.02
**S03**		F/15	Ptosis		No	ORTHO	Normal OU	0.71		0.38		0.71		3.00		**2.29**
**S04**		F/32	Bulging fontanelle		Yes	XT LE	Normal OU	0.40		0.29		0.39		0.53		**0.14**
**S05**		M /43	NF1 and shaking eyes		No	ORTHO	Normal OU	0.35		0.30		0.35		0.40		0.05
**S06**		F/50	Vomiting and shaking eyes		No	ORTHO	Pallor OU	0.17		0.12		0.16		1.45		**1.29**
**S07**		F/65	Weight loss		No	XT LE	Normal OU	0.22		0.17		0.22		0.98		**0.76**
**S08**		M /79	Eye misalignment		No	XT RE	Pallor WSE	0.43		0.38		0.43		3.00		**2.57**
**S09**		M /94	NF1 and shaking eyes		Yes	XT OD/Nys	Pallor OU	0.39		0.34		0.47		0.55		0.08
**S10**		M /95	Vomiting and ptosis		No	ORTHO	Normal OU	0.24		0.19		0.26		0.66		**0.40**
						**Moderate visual impairment (n=8)**										
**S11**		M/3	Vomiting and eye misalignment		No	XT	Normal OU	1.21		0.57		1.19		1.20		0.01
**S12**		F/8	Shaking eyes		Yes	ORTHO/Nys	Normal OU	0.93		0.56		0.93		3.00		**2.07**
**S13**		F/9	Shaking eyes		No	ORTHO/Nys	Cupping OU	0.91		0.54		0.44		0.95		**0.51**
**S14**		F /10	Shaking eyes		Yes	ORTHO/Nys	Cupping OU	0.79		0.42		0.93		0.93		0.00
**S15**		F/17	Shaking eyes		No	ORTHO/Nys	Pallor OU	0.84		0.66		1.09		1.25		**0.14**
**S16**		F /36	Shaking eyes		No	ORTHO/Nys	Normal OU	0.70		0.65		0.71		0.75		0.04
**S17**		F /40	Shaking eyes		No	ORTHO/Nys	NA	0.80		0.75		0.80		3.00		**2.80**
**S18**		M /54	Vomiting		No	XT RE	Cupping OU	0.65		0.60		0.65		1.15		**0.50**
						**Severe visual impairment (n=7)**										
**S19**		F /18	Weight loss and eye misalignment		Yes	XT RE	Cupping OU	1.30		1.12		1.42		3.00		**1.58**
**S20**		M /20	Shaking eyes		No	ORTHO/Nys	Pallor OU	1.00		0.82		1.03		1.04		0.01
**S21**		M /26	Hypotonia		No	XT	Normal OU	1.31		1.17		1.31		1.37		0.06
**S22**		F /26	Eye misalignment		Yes	ET/Nys	Pallor OU	1.16		1.02		1.17		1.27		0.10
**S23**		M /33	Shaking eyes		No	XT LE/Nys	Pallor OU	1.04		0.93		1.04		3.00		**1.96**
**S24**		M /37	Shaking eyes		Yes	ET/Nys	Pallor OU	1.14		1.09		1.14		3.00		**1.86**
**S25**		M /42	Vomiting		No	ORTHO/Nys	Normal OU	1.50		1.45		1.50		3.00		**1.50**

IAD= interocular acuity difference; BSE= better-seeing eye; ET= esotropia; LE= left eye; NF1= neurofibromatosis type 1; Nys= nystagmus; OU= both eyes; RE= right eye; XT= exotropia; WSE= worse-seeing eye. IAD >0.10 logMAR are shown in bold.

Visual abnormalities at diagnosis were reported by parents in 17 children (68.0%) and included shaking eyes (n=12), eye misalignment (n=2), proptosis (n=1), and ptosis (n=1). At SVEP evaluation, nystagmus (n=17; 68.0%), optic disc cupping and/or pallor (n=13; 52.0%), strabismus (n=12; 48.0%), and poor visual behavior (n=9; 36.0%) were observed.

**Figure 2:**
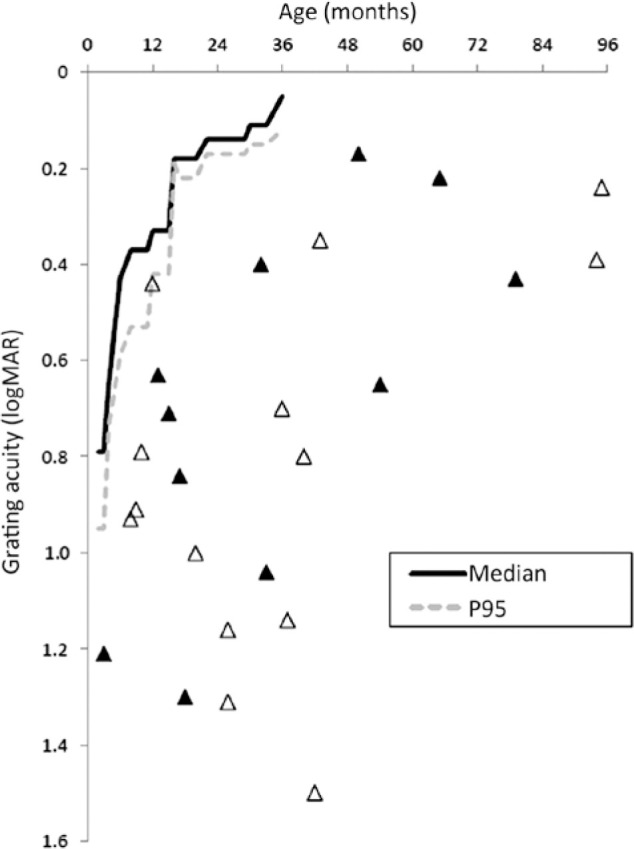
Binocular grating visual acuity scores (in logMAR units) from 25 children with optic pathway tumors compared with age norms from our own laboratory. Boys are represented by black triangles.

**Figure 3:**
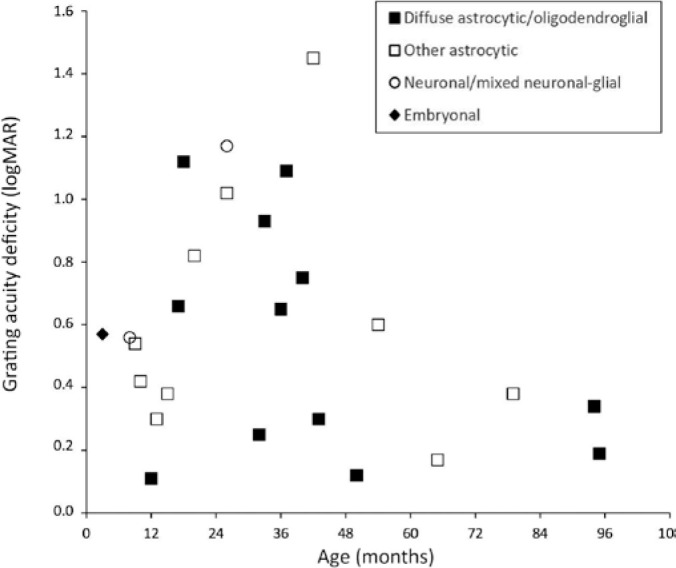
Distribution of 25 children with optic pathway tumors considering World Health Organization classification^(19)^, age at sweep visually evoked potential (SVEP) evaluation (in months), and binocular grating acuity scores (in logMAR units).

Visual impairment based on binocular GVAD (mean ± SD, 0.60 ± 0.36 logMAR; median, 0.56 logMAR) was detected in all children and was classified as mild in 10 children (40.0%), moderate in 8 children (32.0%), and severe in 7 children (28.0%). Increased IAD (>0.1 logMAR) was found in 16 children (64.0%). GVAD was comparable in boys and girls, and no correlation was found between age at tumor onset and GVAD. A representative SVEP response from the BSE and the WSE and the orbital magnetic resonance imaging (MRI) from a participant (subject S01) are shown in figure 1. The binocular GVAD scores, in logMAR units, of each participant in comparison with age norms from our laboratory are shown in figure 2. The distribution of all participants, considering the World Health Organization (WHO) classification of tumors, age (months) at SVEP evaluation, and GVAD scores (in logMAR units), is shown in figure 3.

## DISCUSSION

Visual impairment was determined in a group of young children with hypothalamic-chiasmatic tumors who were unable to perform recognition VA tests. Agebased grating acuity deficits and interocular acuity differences were in line with ophthalmological features and neuroimaging and demonstrated the negative impact of tumor lesions on visual status in all participants. Structural damage to the optic pathway was attributed to inward tumor growth and/or secondary compression by raised intracranial pressure.

A limitation of this study is that the majority of the children had been referred to our emergency service with serious life-threatening clinical conditions, preventing grating acuity measurement before oncologic management.

An important aspect to be considered when dealing with neuropediatric disorders affecting the visual pathway is the critical period of visual cortical development, occurring between birth and approximately 8 years of age^(12,13)^. During this specific window, uninhibited visual inputs from each eye are essential for the development of both monocularand binocular-driven cells in the occipital lobe. Abnormal visual input or disuse of an eye can lead to decreased vision, which should be treated to prevent permanently affected vision^(8)^. Increased interocular acuity differences were observed in 16 of 25 children (64%), including 8 of 12 children with strabismus. According to clinical data (Table 3), eight children with strabismus presented with increased IAD, but only three of them (S01, S08, and S19) had noticeable eye misalignment on tumor diagnosis. Overall, strabismus was reported in the remainder of strabismic children (n=9/12) during follow-up. Thus, in our sample of children affected by optic pathway tumors during the critical period, grating acuity deficits and increased IAD highlight the deleterious impact of optic pathway tumors on vision and binocular interaction, as suggested by ocular motility disturbances.

The proportion of visual disturbances in the current series (Table 4) was similar to those observed in previous reports^(2,6,7,10,21-29)^. Optic disc abnormalities were attributed to axonal losses, whereas strabismus indicated oculomotor pathway disruption or cranial nerve palsies^(8,16)^. Reduced VA may be explained by abnormal information processing in early visual areas^(16)^, pre-existing damage in the visual system, and tumor growth confined to adjacent neural structures without the direct involvement of visual pathway axons^(8)^.

**Table 4:** Summary of studies on visual abnormalities in children with brain tumors, including findings from the current study

Author, year, and location	*N*	Age range (years)	Tumor location	Visual abnormalities at diagnosis
**Wisoff et al., 1990, USA^(23)^**	16	0.2-21.0	Supratentorial, midline, and infratentorial	LVA(n=11) VF loss (n=6) Strabismus/nystagmus (n=4)
**Suharwardy & Elston, 1997, UK^(22)^**	17 (12 boys)	1.0-13.0	Supratentorial, midline, and infratentorial	LVA(n = 16) VF loss (n=9) Optic disc atrophy *(n=7)* Papilledema *(n=4)* Swollen disc (n=3) Disc pallor *(n=2)* Afferent pupillary defect (n=9) Proptosis (n=3)
**Grabenbauer et al., 2000, Germany^(20)^**	25 (14 boys)	1.5-16.0	Optic pathway	Visual disturbances (n=17) Strabismus *(n=5)* VF deficits (n=20)
**Baroncini et al., 2007, France^(21)^**	16 (9 girls)	2.4-14.9	Thalamic	Visual dysfunction *(n=7)*
Crawford et al., 2007, USA^*^24^^’	30 (21 boys)	6.0-17.0	Supratentorial, midline, and infratentorial	LVA(n = 13) Suprasellar: bitemporal hemianopsia *(n=4)* Pineal: 1 upgaze paralysis (Parinaud syndrome) Disseminated: mixed VF defects (n = 1)
Santamaria, 2008, Spain^*^25^^’	58	<14.0	Supratentorial, midline, and infratentorial	Papilledema (n=17) Optic atrophy (n = 14) Nystagmus VF loss Pupillary alterations (n = 18) Dyschromatopsia (n=4) Amaurosis (n=3); Legal blindness (n = 3)
**Wilne et al., 2011, UK^(1)^**	139 (82 boys)	0.8-16.7	Supratentorial, midline, and infratentorial	Papilledema (n = 50) Nystagmus (n = 25) Reduced visual acuity (n=20) Squint (n=18) Diplopia (n=18)
Pillai et al., 2012, Canada^*^7^^’	35 (18 boys)	0.0-1.0	Supratentorial, infratentorial	Sunset eyes *(n*=21) Nystagmus (n=3)
**Ghodsi et al., 2015, Iran^(26)^**	(20 boys)	-1.0	Supratentorial (n = 11) Infratentorial (n=6)	Supratentorial: sunset eyes *(n=2),* nystagmus *(n=2),* visual loss/no pursuit *(n=2),* ptosis (n = 1) Infratentorial: sunset eyes (n = 5), abducens nerve palsy (n=3)
**Hoffmann et al., 2015, Germany^(27)^**	411	0.0-9.0	Sellar/suprasellar	Visual impairment (n=161) Papilledema Optic atrophy
**Alswaina et al., 2015, Saudi Arabia^(2)^**	26 (14 boys)	0.1-17.0	Supratentorial, midline, and infratentorial	LVA with disc pallor (n = 10) LVA with disc swelling (n=3) Acquired strabismus with disc pallor or swelling (n=4) Acquired esotropia with diplopia (n = 3) Acquired exotropia (n=4) Nystagmus (n=3)
**Sánchez-Sánchez et al., 2016, Mexico^(28)^**	51 (28 boys)	0.0-15.0	Supratentorial (n = 19) Infratentorial *(n* = 32)	Nystagmus *(n = 6* ST) Double vision (n = 2 ST + 3 IT) LVA (n =4 ST) Strabismus *(n=2* ST) Papilledema (n=1 ST + 1 IT)
**Stocco et al., 2017, Italy^(29)^**	75 (45 boys)	3.3-11.7	Supratentorial, midline, and infratentorial	Strabismus (n = 16) Abnormal optic disc (n=11) Nystagmus (n = 11) Double vision (n = 9) Papilledema *(n=8)* Visual field defects *(n=7)* Palpebral ptosis (zz=6) Anisocoria *(n=4)* Abnormal pupillary light reflex (n=3) Blurred vision (n = 3) “Eyes wide open” episodes (n = 3) Loss of stereopsis *(n=*1) Abnormal eye movements *(n =* 1) Sunset eyes (n = 1) Visual loss (n = 1)
**Dotto et al., 2020, Brazil^(5)^**	25 (13 boys)	0.2-7.9	Supratentorial/midline	Abnormal grating acuity (n=25) Nystagmus (n = 17) Enlarged 1AD (n=16) Abnormal optic nerve (n=13) Strabismus (n = 12) Abnormal visual behavior (n=9)

IAD= interocular acuity difference; IT= infratentorial; LVA= low visual acuity; ST= supratentorial; VF= visual field.

To hasten the diagnosis of brain tumors in children, a consensus statement from 120 health care providers and parents presented several recommendations related to timely diagnosis. Initial management should include imaging after 2 weeks of persistent visual changes^(30)^. Subsequent evaluation of visual function by SVEP would be extremely relevant to detect visual deficits in uncooperative, preverbal, and nonverbal children suspected of having a brain tumor.

In conclusion, in children with optic pathway tumors who are unable to perform recognition acuity tests, it is possible to detect and quantify visual impairment by objective grating acuity measurement. Detection of grating VA deficits complements clinical investigation and supports the neuro-oncologic management of these conditions.
